# Sports-Related Dystonia

**DOI:** 10.5334/tohm.670

**Published:** 2021-12-21

**Authors:** Abhishek Lenka, Joseph Jankovic

**Affiliations:** 1 Department of Neurology, Medstar Georgetown University Hospital, Washington, District of Columbia, USA; 2 Parkinson’s Disease Center and Movement Disorders Clinic, Department of Neurology, Baylor College of Medicine, Houston, Texas, USA

**Keywords:** Dystonia, sports, task-specific, yips

## Abstract

**Background:**

Task-specific dystonia (TSD) is a form of focal dystonia that occurs in the context of the performance of selective, highly skilled, often repetitive, motor activity. TSD may be apparent during certain tasks such as writing, playing musical instruments, or other activities requiring fine motor control, but may also occur during certain sports, and maybe detrimental to professional athletes’ careers. Therefore, sports physicians and movement disorder neurologists need to be aware of the presentation and phenomenology of sports-related dystonia (SRD), the topic of this review.

**Methods:**

A broad PubMed search using a wide range of keywords and combinations was done in October 2021 to identify suitable articles for this review.

**Results:**

Most of the publications are on yips in golfers and on runners’ dystonia. Other sports in which SRD has been reported are ice skating, tennis, table tennis, pistol shooting, petanque, baseball, and billiards.

**Discussion:**

Yips, which may affect up to half of the golfers and rarely athletes in other sports (e.g., baseball, cricket, basketball, speed skating, gymnastics) seems to be a multi-factorial form of TSD that is particularly troublesome in highly skilled professional golfers. Runners’ dystonia, affecting the foot, leg, and hip (in decreasing order), may evolve into more generalized and less specific dystonia. The pathophysiologic mechanisms of SRD are not well understood. Botulinum toxin has been reported to alleviate dystonia in golfers’, runners’, and other forms of SRD. Future studies should utilize neurophysiologic, imaging, and other techniques to elucidate mechanisms of this underrecognized group of movement disorders.

## Introduction

Dystonia is a movement disorder characterized by sustained or intermittent muscle contraction resulting in abnormal, often repetitive and patterned, movements and/or postures [1]. An international consensus committee has proposed a bi-axial classification scheme for dystonia [1]. Clinical characteristics of dystonia (axis-1 of the classification) may be described under several key aspects such as age at onset (infancy, childhood, adolescence, early adulthood, and late adulthood), the pattern of body involvement (focal, segmental, multifocal, generalized, and hemidystonia), disease course (static or progressive), and variability (persistent, action-specific, diurnal, paroxysmal). Dystonia may be observed in association with other neurological signs or in isolation. Task-specific dystonia (TSD) is a form of isolated focal dystonia that emerges in the context of the performance of a particular, selected motor activity [1,2]. TSD has been reported in association with a wide range of tasks ranging from apparently simple tasks such as writing (writers’ cramp) [3], speaking [4], and running [5] to tasks requiring highly skilled movements. The latter include dystonia associated with playing musical instruments [6,7], typing [8], singing [6], dancing [9], and other performance-related activities such as sports [10]. This review will focus on sports-related dystonia (SRD), a form of TSD.

A growing number of reports have drawn attention to SRD over the last few years and these publications have highlighted the importance of prompt diagnosis and treatment of this type of dystonia. Yips is the most frequently reported SRD which affects up to half of the professional golfers. The term “yips” has been primarily used to describe motor disturbances in golfers while executing shots that require fine motor control (e.g., chipping and putting) [11]. In addition to golfers, this multi-factorial form of TSD which emerges in the context of focal dystonia and choking (described in detail below) rarely affects athletes in other sports such as baseball, cricket, basketball, speed skating, and gymnastics [11]. If left untreated, SRD may become not only troublesome but also ultimately detrimental to the athletes’ professional careers. For example, because of a phenomenon similar to TSD associated with sports, named “twisties” (impaired sense of position in space), one of the premier American gymnasts had to withdraw from the recently concluded Olympic Games in Tokyo [12]. In a case series of 19 athletes with dystonia, a variety of wrong diagnoses were made (piriformis syndrome, claudication, muscular dystrophy, compartment syndrome) prior to the correct diagnoses [13]. Hence, it is important to be aware of SRD as it would not only prevent unnecessary diagnostic procedures but also facilitate early and appropriate management.

In this article, we comprehensively review the phenomenology, pathophysiology, and treatment of various SRD.

## Method of Literature Search

We used PubMed to search the literature for the key aspects of this article. The search was done in October 2021, and we screened abstracts pertinent to the topic of this review published till the date of the literature search (i.e., no filter was set for the publication date). A number of carefully selected keywords representing various sports were combined with dystonia/task-specific dystonia/yips/twisties. We used the advanced search builder in the PubMed to create a Boolean phrase as below- *((((((Dystonia) OR (yips)) OR (spasm)) OR (jerk)) OR (tremor)) OR (movement disorder)) AND ((((((((((((((((((((((sports) OR (golf)) OR (running)) OR (shooting)) OR (tennis)) OR (basketball)) OR (baseball)) OR (table tennis)) OR (archery)) OR (petanque)) OR (cricket)) OR (carrom)) OR (billiards)) OR (badminton)) OR (darting)) OR (soccer)) OR (snooker)) OR (cycling)) OR (fencing)) OR (gymnastics)) OR (skating)) OR (hockey))*. This yielded a total of 8161 articles. We excluded the articles (n=1988) which were not published in the English language and those which were based on non-human subjects. Abstracts of the remaining 6173 articles were carefully screened for case reports, case series, or any original studies reporting any of the SRD. The reference section of the articles was thoroughly examined for additional articles pertinent to the topic of the current review. Eventually, 55 articles were selected for this review (supplementary figure).

A majority of the publications on SRD have been on yips in golfers and lower limb dystonia in runners. Other sports in which TSD has been reported include tennis, table tennis, baseball, basketball, billiards, bowling, pistol shooting, and petanque, in which boules/balls are directed towards a target ball. As described earlier, although TSD is the common substrate of all the aforementioned conditions, the term “yips” has been primarily used for TSD in golfers. In this review, we will not discuss other sports-related movement disorders, such as parkinsonism associated with chronic traumatic encephalopathy which is encountered most frequently in professional football players, pugilistic parkinsonism in boxers [14], or dystonia after central or peripheral sports-related injury [15,16].

## Yips in Professional Golfers

Golf is a sport that requires a high degree of precision, and it challenges the gross motor as well as fine motor skills of golfers. Yips is a form of motor disturbance that may impair performance in amateur and professional golfers. This phenomenon hampers the precise execution of golf strokes which demand fine motor skills (e.g., “putting”). Although golf originated 500-600 years ago in Scotland, it was not until 1977 when the first report of yips was published and it was described as “putting agony” [11,17]. The career of several professional golfers has been threatened or terminated as a result of yips. This term “yips” is believed to be popularized by Tommy Armour, a professional golfer, who described it as a motor impairment during putting which made him retire early from golf [11]. Based on his experience and description (“once you’ve had ‘em, you’ve got ‘em”), it appears that the yips are largely persistent without much hope for spontaneous remission. It is also often referred to as “golfers’ cramp”. In addition to golfers, yips has been described in baseball pitchers, cricket players, basketball players, speed skaters, gymnasts, and other athletes.

### Epidemiology of yips in golfers

There is limited epidemiological data on yips in golfers. So far, only five questionnaire-based studies have attempted to explore the prevalence of yips among professional golfers. The first study, published in 1989, used a 69-item questionnaire which was designed based on the experience of a professional golfer who suffered from the yips [18]. The response rate was 34.3% and among the responders, 28% reported having experienced yips in the past, with almost half reporting bilateral hand involvement [18]. There was a mean latency of 20.9 years (time between the individuals started playing and the emergence of yips). Later, in another questionnaire-based study (response rate of 39%), 53.5% of the golfers reported experiencing yips during their career [19]. However, after analyzing the data only of the golfers with a low handicap index (<10), the prevalence was 25%. Handicap index is a score which indicates the skill level of a golfer (lower the score, better the skill); “scratch player” refers to a highly-skilled golfer with a handicap of zero. These two studies showed that yip was more prevalent in older and experienced golfers who often reported associated anxiety (‘choking’). In another questionnaire-based study with a better response rate (92%) than the aforementioned studies, 39% reported that they experienced yips sometime during their career [20]. Interestingly, a multivariate regression analysis suggested that in addition to a longer golf career, a history of musculoskeletal injuries was an independent risk factor for the emergence of yips. This raises the possibility that some yips could represent peripherally-induced movement disorders [16].

As self-reported yips may not always represent yips and are subject to recall bias, it is important to obtain prevalence data through video or kinematic analysis. The kinematic analysis deals with the objective assessment of movements of body parts through space and time taking into account several parameters such as linear and angular displacements, velocities, and acceleration. In one study the prevalence of yips based on a questionnaire completed by 1306 golfers was 22.4% (for the entire skill range) but this figure nearly doubled (45.2%) when only data from highly skilled golfers (males with handicap index <10 and females with <12) were analyzed [21]. The investigators then evaluated the golfers during putting experiments (1-meter distance) and, based on a pre-specified kinematic threshold, the estimated prevalence with the objective assessment was 16.7% [21]. The authors calculated the kinematic threshold by using data from the kinematic analyses of non-participating yips-affected golfers (who were previously video-recorded and evaluated by knowledgeable raters). In the most recent study on the epidemiology of yips on 912 professional golfers (response rate 26%), 22% reported suffering from yips [22]. There were several differences between the golfers with and without yips; male sex, smoking history, and family history of yips were more common in the former. Similar to the previous studies, golfers with better skills and with a long history of playing golf were more likely to experience yips. ***Table 1*** summarizes the key aspects of these aforementioned studies.

**Table 1:** Summary of studies on the epidemiology of yips in professional golfers.


STUDY VARIABLE	MCDANIEL ET AL. 1989 [18]	SMITH ET AL. 2000 [19]	KLäMPFL ET AL. 2015 [21]	GON ET AL. 2021 [20]	VAN WENSEN ET AL. 2021 [22]

**Country**	USA	USA	Germany	Japan	Netherlands

**Mode of recruitment**	Questionnaire (mailed to 1050)	Questionnaire (mailed to 2630)	Questionnaire (accessed online)	Questionnaire (mailed to 1576)	Questionnaire (mailed to 912)

**Eligibility**	PGA, USGA, LPGA	Based on golf skill Men-HCP < 10 Women HCP < 12	Subsample analyzed Men-HCP < 10 Women HCP < 12	PGA (Japan) and KGU members	Members (age > 18yr) of Rosendaelsche golf club, Netherlands

**Response rate (%)**	*34.3% (M: 42%, F: 10%)	39%	29.7%	92.2%	26%

**% affected with Yips**	28%	53.5%	45.2%	39%	22%

**Age (years)**					

**Yips**	50.5	45.2	41.2 ± 14.1	48	61.7 ± 12.5

**No yips**	47.5	47.4	37.1 ± 11.9	47	60.7 ± 12.0

**Golf experience (years)**					

**Yips**	35.6	30.4	17.7 ± 9.7	30	29.5 ± 13.9

**No yips**	31	30.8	12.8 ± 8.8	27	23.5 ± 11.8


**F:** Female, **HCP:** Handicap index, **KGU:** Kansai Golf Union, **LPGA:** Ladies Professional Golfers’ Association, **M:** Male, **PGA:** Professional Golfers’ Association, **USGA:** United States Golf Association. * Data of only males were included for final analysis.

### Clinical features of yips in golfers

Subjectively, many of the golfers with yips have reported several symptoms such as jerks, spasms, and tremors in varying combinations, particularly when putting or chipping. The jerks were the most common symptom (in 49%) in one of the studies [18]. While yips is episodic in the majority of the cases, in some golfers, yips appears with every putting attempt [19]. Golfers with yips had most trouble with short (<4 feet from the hole) putts that were directed downhill. Those with yips had increased electromyogram activity and exerted more grip force than unaffected golfers [19]. Usually, one hand is affected but it is not uncommon to have subsequent bilateral hand involvement [11,18]. In addition to hands, some golfers may develop dystonia of other body parts. For example, some golfers may experience bothersome head-turning (dystonic torticollis) while putting or swinging [11,23]. In a study on 20 golfers with yips, two had reported “freezing” of hand movements while swinging and six golfers had problems with fixating the eyes on the ball while putting [24]. Yips has been reported during the stress of high-pressure tournaments as well as during routine practice.

Alleviating maneuvers, also referred to as sensory tricks or geste antagoniste, are typical features of dystonia [25,26]. A number of alleviating maneuvers have been also reported in golfers with yips; these include crosshanded putting, using a longer putter, changing head position, and altering visual fixation [18,24]. Other than the TSD, golfers with yips usually have a normal neurological examination.

### Etiopathogenesis of yips in golfers

The exact etiology and pathogenesis of yips among golfers have remained elusive. It is believed that underlying anxiety may play a vital role in the emergence of yips. Using multimodal neuroimaging analyses of resting-state functional connectivity, voxel-based morphometry, and tract-based spatial statistics, a study found that TSD was characterized by abnormal recruitment of parietal and premotor cortices that are modality-specific and heteromodal [27].

Several studies have explored the electrophysiologic correlates of yips in golfers. A study involving 20 golfers (10 with yips, 10 without yips) reported that 5 of the 10 yips-affected golfers had co-contraction of the wrist flexor and extensors during putting, and 3 of those 5 were asymptomatic [28]. This was subsequently confirmed by two additional studies [29,30]. These studies provide evidence that focal dystonia is one of the fundamental abnormalities in yips-affected golfers. One group of authors suggested that yips could be related to both dystonia and “choking”, and proposed categorization of yips into- type-1 (related to dystonia) and type-2 (related to choking) [31]. Subsequently, another study reported similar observations i.e., putting difficulty in type-1 yips-affected golfers was due to motor dysexecution in the context of focal dystonia and that in the type-2 group was related to performance anxiety. However, interpretation of these experimental studies is challenging because of the low sample size [32]. Although several studies have attributed yips to choking (type-2) in some patients, this remains controversial as some studies could differentiate yips-affected and yips-free golfers only by putting performance and kinetic parameters, rather than by performance in psychometric tests [33]. Furthermore, in some patients, there is evidence of overlap between the two types of yips, suggesting an intermediate category.

One theory hypothesized that “reinvestment”, defined as the “attempt to consciously control one’s own movement during skill execution by the application of explicit and rule-based knowledge”, may be associated with the emergence of yips [34]. However, a study that specifically investigated the role of reinvestment in 19 yips-affected golfers did not find any evidence to suggest a link between reinvestment and yips [35].

### Treatment options

It is now generally accepted that focal dystonia is one of the major underlying components of yips in golfers which may or may not be exacerbated by psychological abnormalities. Therefore, treatment of focal dystonia is a reasonable strategy in yips-affected golfers with obvious dystonia [27]. In a previously published report by one of the authors of this article (JJ), two golfers with yips had a clinically gratifying response to botulinum toxin [11]. There is growing evidence that botulinum toxin injection into carefully selected muscles is the treatment of choice for TSD including yips [36,37]. Additionally, oral anti-dystonia medications such as benzodiazepines, trihexyphenidyl hydrochloride, and baclofen may be considered, when appropriate [38]. In very severe cases, pallidal deep brain stimulation (DBS) may be considered. Pallidal DBS was reportedly beneficial for a golfer with severe task-specific cervical dystonia [23]. There are reports of improvement in yips symptoms in golfers after acupuncture [39] and memantine [40] (for concurrent cognitive impairment). However, in the absence of controlled studies, it is difficult to interpret the reported efficacy of such treatments. Several non-pharmacological approaches have been recommended by golf coaches (*https://www.youtube.com/watch?v=PBjtR3sm_Q8*). These include modification of the golfers’ gripping technique, switching to a longer putter, use of hypnosis, etc. [11]. However, none of these have been assessed by appropriately designed controlled trials.

To summarize, yips affects a relatively high number of professional golfers (22% to 53.5%) and the prevalence is greater in highly skilled golfers with a long professional career. Yips may present with several symptoms such as jerks (most common), spasms, and tremors in various combinations. While focal dystonia is implicated in the majority of golfers with yips, underlying anxiety also plays a vital role in the emergence of yips. Treatment of the focal dystonia associated with yips by botulinum toxin or oral muscle relaxants and other anti-dystonia medications may improve yips but this may need to be supplemented by behavioral and physical therapy.

## Dystonia in Runners

Due to the relative rarity of runners’ dystonia, most of the published literature on this entity are case reports or case series. Runners’ dystonia, first described in 1996 in five patients, is a form of TSD experienced primarily by long-distance runners [5]. In these individuals, dystonia was initially associated only with running but later it appeared spontaneously and became more continuous and not necessarily related to running. Runners’ dystonia is usually initially unilateral affecting the foot (e.g., inversion, plantar flexion, supination), knee (e.g., buckling, flexion, and extension), and hip (e.g., flexion) in varying proportions; foot and knee involvement being more common than hip involvement [5,41,42,43,44,45] (***Table 2***). Rarely, runners may develop dystonia in other body parts during running. For example, a report described the case of an athlete who used to develop cervical dystonia only while running [44]. The severity of dystonia (neck turning towards the right side) was related to the speed of running. Another report described an athlete who experienced tilting of the neck and trunk towards the left side while running [41]. This occurred only while running on a track in a counter-clock direction.

**Table 2:** Summary of case reports/series on runners’ dystonia.


AUTHORS	CASES	AGE	AAO	SEX	LIMB	CLINICAL FEATURES	ALLEVIATING MANEUVERS	TREATMENT

Wu and Jankovic 2006 [5]	1	40	37	F	L	Foot flexion and knee extension	Walking on heel or walking backwards with partially flexed knee	Carbamazepine

	2	49	40	F	L	Knee buckling, knee extension and hip flexion	Touching the left hip, sitting with back touching a chair, walking on heels	BoNT

	3	58	46	F	L	Foot inversion 20 min After running, knee extension, foot eversion	None	Levodopa

	4	30	20	M	L	Knee extension and foot Inversion	Walking backwards	THP

	5	46	44	M	R	Plantar flexion	None	BoNT

Leveille and Clement 2008 [43]	1	57	55	F	L	Flexion of 2^nd^ to 5^th^ toes and forefoot while Running (later, with walking cycling, and swimming)	Details NA	BoNT

	2	40	30	M	R	Supination of foot with Long distance running	Details NA	BoNT

Suzuki et al. 2011 [41]	1	59	54	M	L	Tilting of trunk and neck to the left while running in counter-clock direction on running tracks	Holding hands over the head, running in clockwise direction, running forwards and imagining that he is running in clockwise direction	Details NA

McClinton and Heiderscheit 2012 [45]	1	56	53	M	L	Left knee flexion resulting in short stride length	walking on beach, pressure on the hip	Clonazepam, BoNT

Ramdhani and Frucht 2013 [42]	1	24	20	F	L	Inversion and plantar flexion	walking backwards	None

	2	33	31	F	R	Sustained foot inversion	walking backwards Taking side steps in both directions	None

	3	27	22	F	L	Inversion and plantar flexion	walking backwards, imagination of walking backwards	None

Lee et al. 2021 [44]	1	51	NA	M	L	Posturing of head towards right and upward deviation of chin while running	None	BoNT


**AAO:** Age at onset, **BoNT:** Botulinum neurotoxin, **F:** Female, **L:** Left, **M:** Male, **NA:** Not available, **R:** Right, **THP:** Trihexyphenidyl hydrochloride.

Similar to other forms of dystonia, alleviating maneuvers (sensory and motor tricks) may be present in TSD associated with running. A wide range of such maneuvers has been reported which include walking backward, walking sidewise, and applying pressure to the hip. In a study referenced above, the patient’s cervical dystonia which occurred only while running in a counter-clockwise direction subsided after running in a clockwise fashion on the track or even only by imagining that he was running in a clockwise fashion [41]. Holding the hands above the level of the head also alleviated dystonia. ***Table 2*** summarizes the reported clinical features and alleviating maneuvers in runners’ dystonia.

Two other studies on runners’ dystonia addressed the phenomenology and natural history of this form of TSD [46,47]. In a retrospective study, the authors analyzed the pattern of lower extremity dystonia in 20 athletes (runners: 13, non-runners: 7) [46]. The diagnosis was delayed by a median of 3.5 years in those with runners’ dystonia. Similar to the cases in a previous report [5], while dystonia initially emerged only in the setting of running, subsequently other activities such as walking also precipitated dystonia. Sensory tricks were present in 5 of the 13 patients. None of the patients had dystonic tremors and none had a family history of dystonia. In a study based on instrumented gait analysis of 13 patients [47], dystonia was unilateral in 10, bilateral in one, and truncal in two patients. Alleviating sensory or motor maneuvers were present in nine patients. Gait analysis revealed excessive knee extension in five, ankle inversion in five, and plantar flexion in four. Nine patients received BoNT and of those, seven had some beneficial effect.

To summarize, runners’ dystonia is probably the second most frequently reported SRD, after yips. This may affect both the proximal and distal parts of the lower extremities (distal > proximal). Dystonia of other body parts such as the neck and trunk are also seen in runners’ dystonia which may improve with certain alleviating maneuvers. Individuals with troublesome runners’ dystonia may benefit from botulinum toxin injections.

## Dystonia in Other Sports

In addition to golf and running, dystonia has been reported in other sports. Those sports include tennis [48], petanque [49], pistol shooting [50], table tennis [51,52], billiards [53,54], baseball [55], and ice-skating [56]. There is a limited number of articles on dystonia or other movement disorders in these sports, probably because of the rarity of the condition or probably because it is underrecognized or underdiagnosed. ***Table 3*** summarizes the published reports on dystonia related to these sports.

**Table 3:** Summary of reports on dystonia in sports other than golf and running.


AUTHOR	AGE/GENDER/SPORT HANDEDNESS	MANIFESTATION	ALLEVIATING MANEUVERS	IMPAIRMENT OF OTHER ACTIVITIES	TREATMENT

Mayer et al. 1999 [48]	34/M/tennis/L	Abrupt impairment of movements in the cocking phase, interrupting adducting movements while serving, unable to lift the racquet behind the head	None	Writing	BoNT, THP

Le Floch et al. 2002 [52]	21/M/table tennis/R	Involuntary elbow flexion while serving. Elevation and adduction of shoulder while serving and while executing forehand strokes	Yes	Daily activities requiring elbow flexion	Not mentioned

Lagueny et al. 2002 [49]	52/M/petanque/R	Freezing of shoulder flexion while throwing boule at a target	None	None	Not mentioned

	56/M/petanque/R	Freezing of shoulder flexion while throwing boule at a target	None	None	Not mentioned

Sitburana and Ondo 2007 [50]	64/M/pistol/R	Right forearm tightness and hand twisting after holding the pistol (exam- pronation and flexion of forearm)	NA	None	BoNT

Asahi et al. 2016 [51]	20/F/table tennis/L	Flexion of left wrist, first with forehand and later with backhand shots	None	Elevation of left elbow while using chopstick or while operating cell phone	VO thalamotomy

Smilowska et al. 2018 [53]	57/M/billiards/R	Right upper limb stiffness, Anti-flexion of shoulder, Movement arrest	Yes	None	BoNT

Lee et al. 2021 [54]	52/M/billiards/R	Feeling of arm being “locked” while trying to strike a ball extending the the elbow. EMG showed co-contraction of deltoid and triceps immediately after the bicep contraction when pulling the cue backward	None	None	BoNT (poor response)

Nakane et al. 2018 [55]	15/M/baseball/R	Anteflexion and stiffness of Right shoulder. Elbow Flexion associated with adduction and elevation of the shoulder	Yes	Activities requiring the movement of right upper extremity	MAB

	41/M/baseball/R	Jerking of right shoulder, bending and moving of right upper limb away from the midline	Yes	None	BoNT


**BoNT:** Botulinum neurotoxin, **EMG:** Electromyogram, **F:** Female, **L:** Left, **M:** Male, **MAB:** Muscle afferent block, **R:** Right, **THP:** Trihexyphenidyl hydrochloride, **VO:** Ventrooralis.

Yips in the Dutch ice speed skaters was reported in a series of five subjects by a group of authors led by Nijenhuis, a former Olympic speed skater who was affected by yips [56]. In the Netherlands, this skaters’ cramp is known as “zwabbervoet” (English- mop foot) and it has hampered the professional career of several Dutch skaters. The skaters usually describe this as a feeling of reduced control in the affected foot/leg which occurs only while speeding and inline skating. Visually it may manifest as a twitch or jerky spasm when the skate is suspended in the air and before it is placed on the ice (in the swing phase) [56]. In the above-referenced study, three out of the five skaters experienced extreme stress prior to the onset of the yips. Interestingly, unlike yips in golfers, the skaters experienced sensory abnormalities such as numbness and reduced position/vibration sensation in their legs. Video analysis revealed muscular jerking in all patients immediately before, during, and after skate placement. Kinematic analysis revealed that angular velocity in the affected leg of the skaters was much higher than that in the unaffected leg. Overall, this study highlighted the role of TSD in ice skaters with yips.

## Conclusion and Future Perspectives

SRD may become detrimental to the performance of athletes and may prematurely terminate the career of sports professionals. As emphasized earlier, familiarity with the SRD is critical in preventing unnecessary investigations and in facilitating the earlier diagnosis of this debilitating condition, leading to the earlier implementation of suitable treatment. Future studies should focus on longitudinal follow-ups to determine the natural history of SRD and should use kinematic analysis and other neurophysiologic techniques to better understand this complex phenomenon. A better understanding of the pathophysiology of SRD may lead to more effective therapies.

## Additional File

The additional file for this article can be found as follows:

10.5334/tohm.670.s1 Supplementary Figure. Summary of the literature search in PubMed.
