# Supplementary figures and images for: Sports-Related Dystonia
Source: Tremor Other Hyperkinet Mov (N Y). 2021 Dec 21;11:54. doi: 10.5334/tohm.670 (PMC8698216; doi:10.5334/tohm.670)

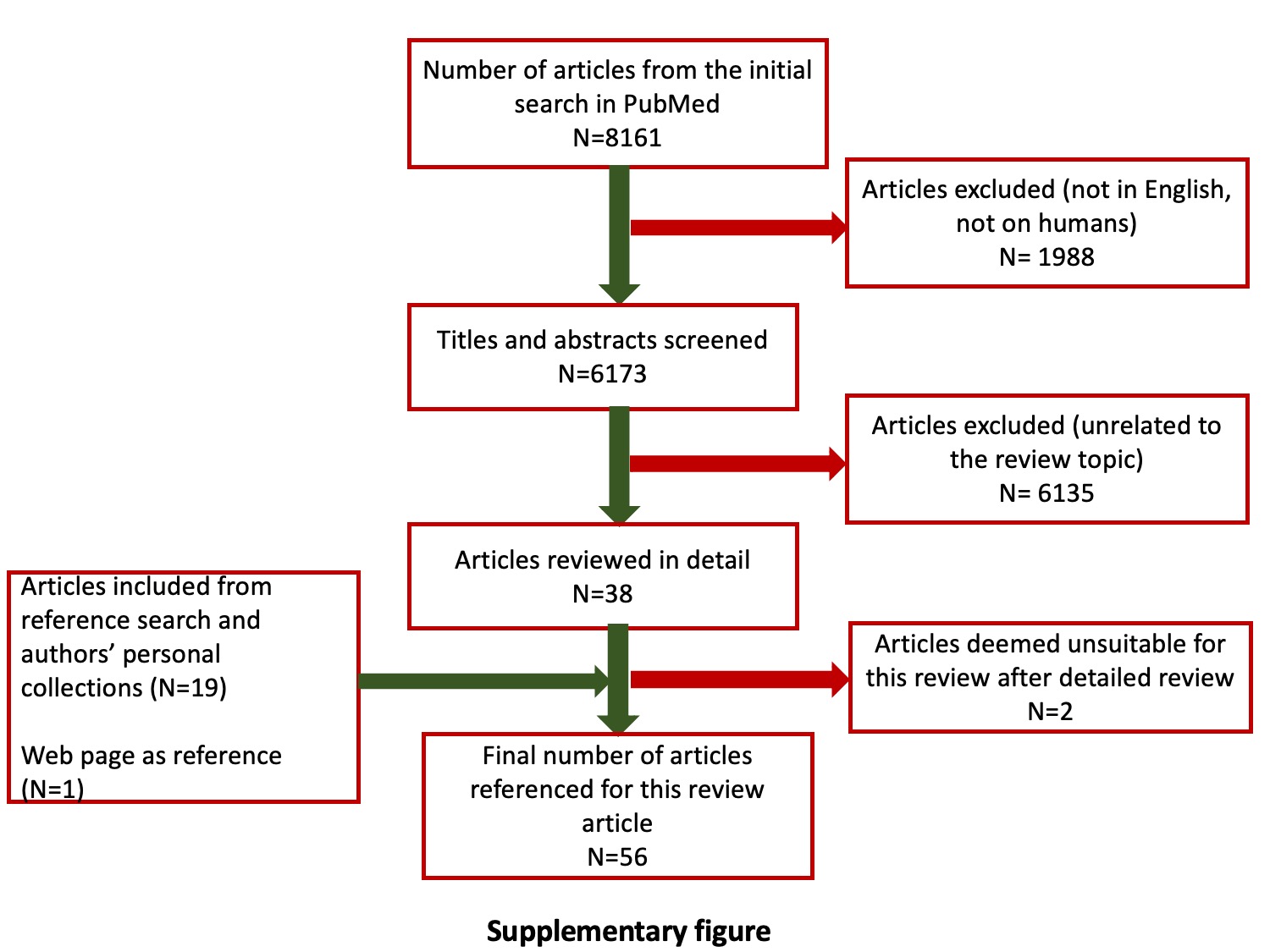

Supplement: Supplementary Figure. — Summary of the literature search in PubMed. [file tohm-11-1-670-s1.jpg]
